# Antifebrin in the Treatment of Epilepsy

**Published:** 1889-08

**Authors:** Theodore Diller

**Affiliations:** Danville, Pa.


### ANTIFEBRIN IN THE TREATMENT OF EPILEPSY.

BY THEODORE DILLER, M. D., DANVILLE, PA.

Nine cases of epilepsy in this hospital in which the treatment by antifebrin was inaugurated were not in the least selected, but were simply taken in a haphazard way. As a matter of fact, they represent a wide dissimilarity in many respects; and it has seemed to me that a group of cases which would comprise as many phases of the disease as possible would be the best suited upon which to test the efficacy of a new therapeutic agent. The fact must not be lost sight of, however, that while these cases present important differences, yet they are all alike, in that each one has a degree of psychical disturbance which is considered by two reputable physicians to be of such nature as to requtre detention in a hospital for the insane. The drug was given, with more or less interruption, during a period of about four and a half months,—viz., during part of October, and all of November, December, January and February. For greater convenience in calculation, and because of the possibility that the effects of the remedy were not fully established until after it had been administered a short time, I consider that the drug has been taken only during the months of November, December, January, and February last; but, in comparing number of fits occurring in these months with those which took place in previous months, it would be obviously unfair to include the month of October in either list. So, in drawing comparisons, this month is omitted from consideration.

The drug was administered three times a day, dry, upon the tongue. Dose, 5 grains, except in cases 3 and 4, where this amount was doubled. As the powder is tasteless and practically insoluble, this mode of administration, I think, will generally be found to be the best. Some patients, however, might prefer capsules, and, of course, the dose could easily be given in this way.

For obvious reasons, the only observations which could be made in the space of four months of the effects of any agent in the treatment of epilepsy, would be upon the frequency and character of the fits, and to a less extent, upon the general physical, mental, and moral *status* of the patieut. Any estimate of the drug as a *curative* agent, based upon such meagre data, would be immature and unprofitable.

I will as briefly as possible cite the cases, all females, and make comments upon them afterwards.

Case 1. Aged 29; admitted 1884. Case of nine (?) years' standing; dark hair and eyes; rather undersized woman; slight figure; fair physical health; intellect very dull. Sits quietly in one place all day. No history of head injury, but large cicatrix noted just back of left frontal eminence, probably result of an old burn.

Case 2. Aged 16; stupid, expressionless face; silly, unmeaning grin. At times she is quite cyanotic; comprehends only simplest ideas; unable to attend to personal wants. General health poor. Refused medicine very frequently.

Case 3. Aged about 40. Fits very violent. She is nervous and uncertain in her movements. Moderately bright at times. Has periods of violent excitement after a fit.

Case 4. Mental power very limited. Silly in manner, and, after a series of fits, seems to be for a time totally unconscious of her surroundings. Fits can be controlled by the use of bromides, but this treatment has a marked depressing effect, both mentally and physically.

Case 5. Aged 34. Laborer's wife; has had epileptic fits for past nineteen (?) years. Mother is an epileptic. Fits of *grand-mal* type; often nocturnal; always violent. Patient reads, sews, etc. She is pretty bright mentally, but apt to complain of trivial matters.

Case 6. Aged 43; single; average height; slight figure; blonde; admitted 1884. History says she has from four to eight fits per week. Acts in a silly, child-like manner. Fits very violent.

Case 7. Aged 34; single; bright and ladylike ordinarily, but easily depressed in spirits; has violent outbursts of temper; unstable in character. Fits of *grand-mal* variety and without an aura.

Case 8. Aged 42; married. Tall, thin woman; bilious temperament; stupid and morose. Sews and does light work pretty well; has periods of very violent excitement, which last several days. After they have subsided she has no remembrance of them.

Case 9. Aged 13; admitted 1887; very dull and stupid; gets fits in series; at times in the *status epilepticus*. Before actual onset of such a period she is usually much excited, tears clothing, throws any article she can out the window. Obstinate; apt to pick skin into open sores with pin. General health is poor.

Cases 1 and 5 took the antifebrin during the four months continuously, except two weeks in December. The administration of the drug in the remainder of the cases was interrupted to a greater or less extent. This because of refusal to receive the medicine, mental excitement, etc.; but from December 2 to 14 the treatment was in all cases suspended because of a failure of the supply of the drug.

The remedy appeared to be well borne in all cases. The mental and physical depression and gastric disturbance observed so often in the bromide treatment were not apparent. It will be observed that the greatest decreases in the number of fits under the antifebrin treatment are in cases 1 and 5. There is less of a decrease in cases 3, 4, 6 and 8, while in the remaining cases—2, 7 and 9, there is a slight increase. Of this last group, however, it may be said that the remedy did not have a fair chance, as it was so much of the time refused by the patient or set aside for a tonic treatment.

In cases 2 and 3 no fits occurred in the month of September, and in case 4 only one fit in each of the months of June and July. This while the patient was under the influence of the bromide treatment. It shows well the strong controling influence of the treatment when we compare the records with those of months immediately before or after the months mentioned. It will be noticed that no such marked contrasts are recorded under the antifebrin treatment. The antifebrin did not succeed in any one case, having an average of over ten fits a month, in completely abolishing the attacks during a single month, as was the case under the bromide treatment. Yet in the two cases (1 and 5) responding best to the antifebrin treatment the seizures were pretty uniformly diminished in number each of the four months this treatment was pursued. This is also, though in less degree, true of the remaining five cases, particularly if we leave out of consideration cases 2 and 9.

To conclude, it may be said:

1. That in all the cases in which the drug was given continuously there was noted a reduction in the number of fits, ranging from about twenty-five to seventy-five per cent., as compared with other months during which patients were on bromide and tonic treatment alternately.

2. The remedy was in all cases well borne, producing no apparent mental or physical depression. This in marked contrast with depressant effects noted after a course of bromide treatment.

3. No skin eruption was produced.

4. In any given case, in which a great number of fits are occurring, and where it is desirable to control them as soon as possible, the bromides would be of far more value than antifebrin.—*Therap. Gaz.*

